# Clinical development of molecular residual disease (MRD) and multi-cancer early detection (MCED) using liquid biopsy multiomics with artificial intelligence (AI)

**DOI:** 10.1007/s10147-026-03001-6

**Published:** 2026-03-06

**Authors:** Taro Shibuki, Riu Yamashita, Tadayoshi Hashimoto, Takao Fujisawa, Mitsuho Imai, Junichiro Yuda, Takeshi Kuwata, Toshihiro Misumi, Yoshiaki Nakamura, Hideaki Bando, Kaname Kojima, Sayuri Tokioka, Ippei Chiba, Naoki Nakaya, Atsushi Hozawa, Seizo Koshiba, Nobuo Fuse, Sakae Saito, Ritsuko Shimizu, Woong-Yang Park, Kengo Kinoshita, Takayuki Yoshino

**Affiliations:** 1 Department of Hepatobiliary and Pancreatic Oncology, National Cancer Center Hospital East, Kashiwa, Japan; 2 Department for the Promotion of Drug and Diagnostic Development, Division of Drug and Diagnostic Development Promotion, Translational Research Support Office, National Cancer Center Hospital East, 6-5-1, Kashiwanoha, Kashiwa, Chiba 277-8577 Japan; 3 Division of Translational Informatics, Exploratory Oncology Research and Clinical Trial Center, National Cancer Center Hospital East, Kashiwa, Japan; 4 Department of Gastroenterology and Gastrointestinal Oncology, National Cancer Center Hospital East, Kashiwa, Japan; 5 Department of Head and Neck Medical Oncology, National Cancer Center Hospital East, Kashiwa, Japan; 6 Department of Hematology, National Cancer Center Hospital East, Kashiwa, Japan; 7 Department of Genetic Medicine and Services, National Cancer Center Hospital East, Kashiwa, Japan; 8 Department of Data Science, National Cancer Center Hospital East, Chiba, Japan; 9 Tohoku Medical Megabank Organization, Tohoku University, Sendai, Japan; 10 Advanced Research Center for Innovations in Next-Generation Medicine, Tohoku University, Sendai, Japan; 11 GxD Inc., Kashiwa, Japan; 12 Translational Genomics Center, Samsung Medical Center, Seoul, Korea

**Keywords:** Multi-cancer early detection, Cancer screening, Cell-free DNA, Circulating tumor DNA, Multiomics, MCED

## Abstract

**Background:**

Early detection of cancer and precise recurrence monitoring remain major unmet needs in oncology. Conventional screening is limited to a few cancer types, leaving nearly half of cancers without established programs. Multi-cancer early detection (MCED) tests based on circulating tumor biomarkers have shown promise, but sensitivity for early-stage remains a challenge. In parallel, detection of molecular residual disease (MRD) using circulating tumor DNA (ctDNA) has emerged as a powerful prognostic and predictive tool, though current assays remain limited in sensitivity and specificity. This study aims to integrate multi-omics data to develop more refined and highly sensitive MCED and MRD assays.

**Methods:**

This study leverages clinical information and biospecimens from patients with cancer and cancer-naïve individuals. Samples from patients with cancers will be derived from the MONSTAR-SCREEN-3 study, while those from cancer-naïve individuals will be obtained from the Tohoku Medical Megabank Project. Comprehensive analyses will include whole-genome sequencing (WGS), whole-exome sequencing (WES), whole-transcriptome sequencing (WTS), proteomics, metabolomics, and microbiome profiling using stool and saliva. Artificial intelligence (AI)-based multi-omics integration will be performed to develop novel MCED and MRD assays and to evaluate their clinical performance. The primary endpoints are the sensitivity and specificity of MCED and MRD assays.

**Discussion:**

This is the first large-scale study to integrate comprehensive multi-omics profiling with AI for MCED and MRD assay development. The findings are expected to advance precision oncology by improving early diagnosis and recurrence monitoring.

**Trial registration:**

UMIN000053815, approved by the Institutional Review Board of the National Cancer Center Hospital East.

## Introduction

**Fig. 1:**
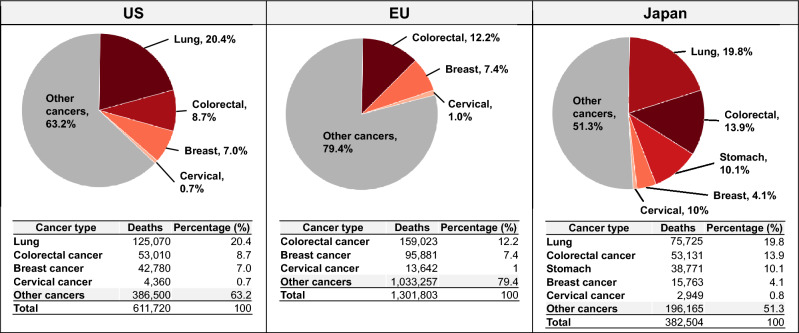
Proportion of cancer deaths attributable to currently screenable cancers versus other cancers in the United States, European Union, and Japan. Population-based cancer screening programs in these regions primarily target a limited number of cancers. As shown, these screened cancers account for less than half of all cancer deaths, while cancers without established screening methods represent the majority of deaths. Abbreviations: *US* the United States, *EU* European Union

Cancer is responsible for approximately 10 million deaths annually worldwide and remains the leading cause of death in Japan, with about one million new cases and 380,000 deaths each year. Patients diagnosed at an early stage have a favorable chance of long-term survival, while those diagnosed at an advanced stage experience markedly reduced survival rates [1, 2]. These findings underscore the critical importance of early diagnosis and prompt therapeutic intervention in improving patient outcomes. Even in developed countries such as the European Union (EU), the United States, and Japan, population-based cancer screening programs are currently established for only a limited number of cancer types, including breast, colorectal, gastric, lung, and cervical cancers. However, these screening methods are often neither simple nor easily accessible, and challenges such as procedural invasiveness and low participation rates are common across regions. Moreover, cancers other than these limited types still account for the majority of cancer-related deaths, representing approximately 63.2% in the United States, 79.4% in the EU, and 51.3% in Japan (Fig. 1) [3–5]. As a result, many cancers are diagnosed at advanced stages, leading to poor outcomes. Therefore, there is an urgent need to develop new, simple, and minimally invasive detection approaches capable of identifying a broader range of cancers at earlier, more treatable stages.

In this context, blood-based analyses using plasma samples have emerged as a promising approach for both early cancer detection and post-treatment disease monitoring. Recently, multi-cancer early detection (MCED) tests that analyze tumor-derived biomarkers in blood, such as genomic alterations, abnormal DNA methylation, and cell-free DNA fragmentation patterns, have been developed as a less invasive and more accessible screening approach. Large-scale prospective studies have demonstrated the clinical potential of MCED testing, revealing its ability to facilitate early diagnosis even in cancer types for which conventional screening methods are unavailable. Although existing studies have targeted different cancer types and employed diverse omics approaches, making direct comparison across studies challenging, the accumulated evidence collectively suggests the clinical utility of MCED as a promising approach for population-level cancer screening. Despite these encouraging findings, challenges remain, particularly the need to improve sensitivity for early-stage cancers. In parallel, the detection of molecular residual disease (MRD) in post-treatment patients has emerged as a promising approach for identifying early recurrence after curative therapy. In particular, MRD analysis using circulating tumor DNA (ctDNA) has been shown to be useful for predicting recurrence risk and treatment response across various cancer types. However, in low-shedding tumors, the sensitivity and specificity of existing assays remain limited, making it challenging to reliably detect minimal residual disease or early relapse.

Thus, while both MCED and MRD hold considerable clinical promise, current technologies still face challenges in achieving sufficient sensitivity and specificity. Most previously reported assays have been based on one or two omics layers, which may not adequately meet these clinical demands. In the present study, by integrating multiple layers of omics data, including whole-genome sequencing (WGS), whole-exome sequencing (WES), whole-transcriptome sequencing (WTS), proteomics, metabolomics, and microbiome, with AI-driven analytical approaches, we aim to develop MCED and MRD assays with greater precision and sensitivity. To ensure the robustness and reproducibility of these analyses, the majority of platforms, workflows, and analytical pipelines have been standardized as much as possible, thereby minimizing batch effects and maintaining data integrity.

This study (UMIN000053815) is conducted on the basis of two large-scale cohort platforms in Japan: MONSTAR-SCREEN-3 study, a nationwide molecular screening project for cancer patients, and the Tohoku Medical Megabank Organization (ToMMo), which maintains comprehensive multi-omics data from the general population. By integrating multi-omics data, the study aims to develop highly sensitive MCED and MRD assays.

## Methods/design

### Study design

This study was conducted utilizing two platforms in Japan: MONSTAR-SCREEN-3 and the ToMMo. These cohorts provide extensive clinical and multi-omics resources that enable analyses for the development of high-performance MCED and MRD assays.

MONSTAR-SCREEN-3 (UMIN000053975), launched in 2024, is a nationwide multi-center project comprising three cohorts (advanced solid tumors, curatively resectable solid tumors, and hematologic malignancies) enrolling approximately 3,200 patients. Through multi-layered omics analyses, including whole-genome sequencing (WGS), whole-transcriptome sequencing (WTS), proteomics, metabolomics, spatial transcriptomics, and microbiome profiling, molecular and clinical imaging data are integrated to develop next-generation AI-based biomarkers.

ToMMo, established in 2012, is one of Japan’s largest biobank and population cohort research initiatives, originally found to support medical reconstruction following the Great East Japan Earthquake and to advance precision medicine. The project has enrolled approximately 150,000 population-based participants, systematically collecting a wide range of biospecimens (blood, urine, saliva, breast milk, and peripheral blood mononuclear cells) and integrating these materials into comprehensive genomic and omics databases for future medical innovation.

**Fig. 2:**
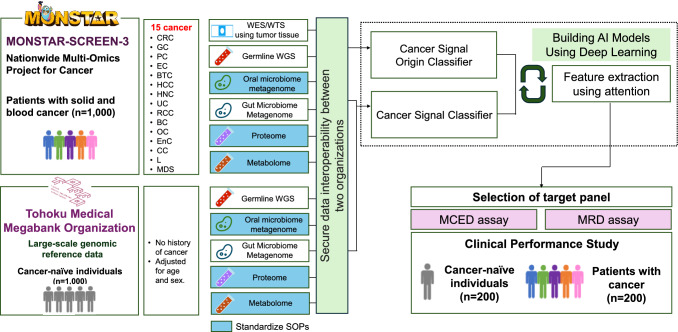
Overview of the study. Using multi-omics data from 1000 cancer patients in the MONSTAR-SCREEN-3 study and 1000 cancer-naïve individuals in the ToMMo cohort, cancer signal origin and cancer signal classifiers will be developed to guide the creation of MCED and MRD assays. Clinical performance studies will then be conducted using samples from 200 cancer patients and 200 cancer-naïve individuals. Abbreviations: *WES* whole-exome sequencing, *WTS* whole-transcriptome sequencing, *WGS*, whole-genome sequencing, *SOP* standard operating procedure, *AI* artificial intelligence, *MCED* multi-cancer early detection, *MRD* minimal residual disease, *CRC* colorectal cancer, *GC* gastric cancer, *PC*, pancreatic cancer, *EC* esophageal cancer, *BTC* biliary tract cancer, *HCC* hepatocellular carcinoma, *HNC* head and neck cancer, *UC* urothelial carcinoma, *RCC* renal cell carcinoma, *BC* breast cancer, *OC* ovarian cancer, *EnC* endometrial cancer, *CC* cervical cancer, *L* leukemia, *MDS* myelodysplastic syndromes

Using existing retrospective clinical and multi-omics data, we will develop AI classifiers for cancer detection and tissue-of-origin prediction based on data from cancer patients in the MONSTAR-SCREEN-3 study and cancer-naïve individuals in the ToMMo cohort. These classifiers will guide the development of MCED and MRD assays, whose clinical performance will then be validated using archived specimens from the MONSTAR-SCREEN-3 study and prospectively collected samples from the ToMMo cohort (Fig. 2).

## Study population

### Development of AI classifiers for cancer detection and cancer-of-origin prediction

This study will first develop artificial intelligence (AI) classifiers to predict the presence of cancer and identify the tissue of origin using multi-omics data obtained from the MONSTAR-SCREEN-3 study and the ToMMo cohort. In total, multi-omics data from approximately 1,000 consecutive cancer patients (MONSTAR-SCREEN-3) and 1,000 background-aligned cancer-naïve individuals (ToMMo) across about 15 cancer types will be analyzed. Up to 1,000 multi-omics datasets from the MONSTAR-SCREEN-3 study, including tumor WGS/WES, WTS, germline WGS, plasma proteome/metabolome, and oral/gut microbiome data, will be analyzed alongside approximately 1,000 background-matched cancer-naïve individuals from the ToMMo cohort undergoing comparable profiling. Clinical information such as age and sex will be retrieved from the ToMMo database. Plasma proteomic analysis will be performed on approximately 1,000 archived plasma samples from the ToMMo biobank using the O-link HT system. All analyses will be conducted with approval from the ToMMo Sample and Data Access Committee. The datasets obtained from both the MONSTAR-SCREEN-3 study and the ToMMo cohort will be integrated to develop deep learning-based AI classifiers, including the Cancer Signal Classifier (detecting the presence of cancer) and the Cancer Signal Origin Classifier (predicting the tissue of origin). Supervised machine learning and deep learning models will be developed and integrated using ensemble techniques. The integrated dataset will be divided into training and internal validation sets to optimize model performance and minimize overfitting. Multi-omics data integration will be employed to identify features associated with cancer presence and tissue of origin. These features will be systematically evaluated to assess their compatibility with ctDNA-based assays. Based on this evaluation, ctDNA-compatible signatures will be selected and used for the development of blood-based MCED and MRD assays. The resulting models are intended to support scalable cancer detection and longitudinal disease monitoring.

### Clinical performance test of MCED and MRD assays

The clinical performance of MCED and MRD assays will be evaluated using approximately 200 samples, each from the MONSTAR-SCREEN-3 study and the ToMMo cohort. In the MONSTAR-SCREEN-3 study, approximately 200 archived plasma samples collected from participants in the main study will be analyzed for ctDNA. In the ToMMo cohort, up to 200 participants will be newly recruited after obtaining written informed consent. In addition to routine ToMMo blood collection (36 mL), an additional 10 mL of blood will be drawn using a ccfDNA PAXgene tube for ctDNA analysis.

### Specimen collection and processing

To minimize batch effects across study cohorts, specimen collection and processing procedures were standardized as much as possible between the MONSTAR-SCREEN-3 study and the ToMMo cohort. For proteomic and metabolomic analyses, blood samples were collected in EDTA-2Na tubes, kept at 4 °C, and centrifuged within 8 h of collection at 2,330 × g for 10 min at 4 °C. The obtained plasma was aliquoted and stored at – 80 °C. For salivary microbiome analysis, saliva samples were collected using Mini centrifuge tubes and stored at – 80 °C immediately after collection. Tumor specimens in the MONSTAR-SCREEN-3 study were collected and stored in accordance with a standardized protocol, ensuring consistency in tissue handling and preservation. WES and WTS analyses of these tumor samples were centrally performed by GxD company, using harmonized pipelines to maintain analytical uniformity. For ctDNA analysis, blood samples were collected using PAXgene Blood ccfDNA tubes. Samples were centrifuged within 7 days after collection: the first centrifugation was performed at 1,900 × g for 15 min at 15–25 °C, followed by a second centrifugation at 1,900 × g for 10 min at 15–25 °C. The separated plasma was stored at − 80 °C until analysis. Although the analytical methods for WGS and fecal microbiome analyses differed between the MONSTAR-SCREEN-3 study and the ToMMo cohort, systematic biases were minimized during AI classifier development by applying standardized feature scaling across datasets.

### Eligibility criteria

Participants who, as of March 2026, are enrolled in the MONSTAR-SCREEN-3 study have not withdrawn consent, and have not declined the secondary use of their clinical data or biospecimens (1). Participants in the MONSTAR-SCREEN-3 study must be 18 years of age or older (2). Individuals who participated between May 2013 and September 2025 in either the ToMMo Three-Generation Cohort Study or the Community-Based Cohort Study, who have not withdrawn consent, and have not refused the secondary use of their data or samples will also be eligible. For participants undergoing additional blood collection for clinical performance testing, written informed consent for this study will be voluntarily obtained (3). ToMMo participants must have no history of malignant tumors (4) and must be between 20 and 75 years of age at the time of consent (5). To ensure background comparability, the age and sex distributions of ToMMo participants will be strictly adjusted to match those of patients with solid tumors enrolled in the previous MONSTAR-SCREEN-2 study (UMIN000043899).

### Exclusion criteria

Participants who are judged by the investigator to lack sufficient decision-making capacity regarding the conduct of this study will be excluded.

### Study period

The enrollment period will extend from February 27, 2024, to September 30, 2026, and the overall study period will continue until March 31, 2027.

### Clinical and multi-omics information

Clinical information (age, sex, cancer type, and clinical stage), as well as multi-omics data obtained from tissue, blood, saliva, and stool samples, including proteomic, metabolomic, and oral/gut microbiome profiles, will be collected.

### Endpoints

The primary endpoint is to evaluate the clinical performance of the developed MRD and MCED assays, including measures such as sensitivity and specificity. The secondary endpoints are to further assess the clinical performance of these assays according to cancer type, clinical stage, and other relevant subgroups.

### Sample size and power

This study is designed for the development and internal validation of AI-based MCED and MRD assays, with sample size considerations based on the precision of performance estimation rather than formal hypothesis testing. The planned sample size of approximately 1000 cancer patients (MONSTAR-SCREEN-3) and 1000 cancer-naïve individuals (ToMMo) is expected to provide adequate statistical precision for model development. Based on preliminary assumptions, the 95% confidence interval (CI) for the area under the curve (AUC) of the cancer detection classifier is expected to range from 0.83 to 0.97 when AUC = 0.85, and from 0.89 to 0.91 when AUC = 0.90, assuming 1000 cancer and 1000 non-cancer samples. For the cancer-of-origin prediction classifier, assuming 50 cases of a specific cancer type and 950 non-cases, the expected 95% CI is 0.78–0.92 at AUC = 0.85 and 0.84–0.96 at AUC = 0.90. These estimates indicate that the planned sample size will ensure sufficient statistical reliability and power for AI classifier development.

### Statistical analysis

To evaluate the clinical performance of the developed MRD and MCED assays, sensitivity, specificity, and their corresponding 95% confidence intervals will be calculated. For continuous variables, summary statistics will be computed, and for categorical variables, frequency distributions will be tabulated. Additional statistical analyses will be conducted as appropriate based on the data characteristics. The performance of AI classifiers will be evaluated using sensitivity, specificity, and AUC with 95% confidence intervals. Internal validation will be used to assess model robustness and generalizability.

## Discussion

This study represents the first large-scale, integrative multi-omics analysis for cancer detection in Japan, utilizing two nationwide cohorts, MONSTAR-SCREEN-3 and ToMMo, to develop highly sensitive MCED and MRD assays. By integrating genomic, transcriptomic, proteomic, metabolomic, and microbiome data through AI-driven modeling, the study aims to overcome the sensitivity limitations of conventional single- or dual-omics approaches and to establish an innovative liquid biopsy platform for both early detection and post-treatment monitoring.

Liquid biopsy-based MCED assays, such as Galleri and CancerSEEK, have demonstrated the feasibility of detecting multiple cancer types using cfDNA methylation, fragmentation patterns, copy number alterations, and circulating protein biomarkers [6, 7]. Across major prospective studies, overall sensitivities range from approximately 27% to 72%, while specificity consistently exceeds 98–99%. However, the sensitivity for early-stage cancers remains limited, typically around 30–50%, underscoring the need for next-generation assays with improved detection capability [6–11]. Similarly, MRD assays have shown promise for detecting molecular residual disease following curative therapy. Tumor-naive assays, which do not require prior tumor sequencing, are more broadly applicable in clinical practice but generally demonstrate lower sensitivity compared with tumor-informed approaches, particularly in early-stage and low-shedding tumors [12]. This challenge highlights the need to develop next-generation MRD assays capable of detecting minimal disease burden with higher sensitivity across diverse clinical settings. The present study seeks to address these needs through an AI-driven, multi-omics integration strategy designed to enable comprehensive and highly sensitive detection of both early-stage and residual disease.

A notable strength of this study is the rigorous standardization of sample collection and analytical workflows across cohorts to minimize technical batch effects, which have historically confounded large-scale multi-omics studies [13, 14]. In our protocol, tissue-derived samples for WTS and WES are collected strictly in accordance with predefined SOPs and analyzed by a single company using a unified sequencing platform. Similarly, proteomic, metabolomic, and microbiome analyses are performed with harmonized logistics and analytical pipelines wherever feasible. This approach not only reduces non-biological variation, but also establishes a robust and harmonized multi-omics dataset that may serve as a reference standard for comparative analyses between cancer and cancer-naïve populations. Such a framework is expected to enhance the robustness, reproducibility, and clinical interpretability of AI-based classifiers.

Furthermore, the integration of multi-omics data through AI-driven modeling embodies a *trans-omics* approach, enabling the capture of complex cross-layer interactions that conventional single- or dual-omics assays fail to resolve. Such interactions may underlie early tumorigenic processes or subtle host–tumor dynamics, thereby enhancing sensitivity for early cancer detection and MRD monitoring.

Moreover, unlike conventional screening programs that focus on a small number of cancer types, this study aims to expand detection to approximately 15 malignancies. By aggregating multiple cancers within a single screening framework, MCED increases the effective disease prevalence, which is a critical determinant of positive predictive value in population-based screening [14]. This conceptual advantage is particularly important for cancers with low individual incidence, such as pancreatic cancer, where single-organ screening approaches are inherently limited. To improve clinical interpretability after a positive result, this study incorporates an AI-based Cancer Signal Origin Classifier to estimate the tissue of origin, thereby supporting more targeted diagnostic follow-up. In addition, while most previously reported MCED studies have focused primarily on solid tumors, our study uniquely incorporates hematologic malignancies within the MONSTAR-SCREEN-3 platform. By capturing both solid and hematologic tumors, this project enables the development of a truly pan-cancer detection framework, further expanding the potential clinical impact of MCED and MRD assays. From a population health perspective, MCED and MRD assays represent a promising strategy to address the substantial and growing societal burden of cancer by enabling earlier detection and shifting treatment toward less intensive and more effective interventions. Earlier diagnosis has the potential not only to improve clinical outcomes, but also to reduce downstream medical costs associated with advanced-stage disease, long-term care, and productivity loss. To ensure sustainable implementation at the population level, however, cost considerations, including potential financial burden for individuals and healthcare systems, should be carefully addressed. This expanded scope has the potential to substantially enhance the clinical utility of MCED and MRD testing, contributing to earlier detection and improved outcomes across a wider range of cancers.

Ultimately, this study is expected to lay the foundation for next-generation liquid biopsy diagnostics that are both highly sensitive and broadly applicable across cancer types. By integrating multi-layered omics data with harmonized protocols and advanced AI analytics, this approach could not only improve early detection and recurrence monitoring, but also guide risk-adapted surveillance and therapeutic decision-making in clinical practice.

## Data Availability

To protect patient privacy and confidentiality, the clinical and activity tracking data from this study are not publicly available. However, de-identified data may be available from the corresponding author upon reasonable request and with appropriate approval from the study steering committee. Any data-sharing requests will be reviewed to verify whether the request is subject to any intellectual property or confidentiality obligations.
